# Natriuretic peptide vs. clinical information for diagnosis of left ventricular systolic dysfunction in primary care

**DOI:** 10.1186/1471-2296-9-14

**Published:** 2008-02-25

**Authors:** Janka Koschack, Martin Scherer, Claus Lüers, Michael M Kochen, Dirk Wetzel, Sibylle Kleta, Claudia Pouwels, Rolf Wachter, Christoph Herrmann-Lingen, Burkert Pieske, Lutz Binder

**Affiliations:** 1 Department of General Practice, Georg-August-University Göttingen, Germany; 2 Department of Cardiology and Pneumology, Georg-August-University Göttingen, Germany; 3 Department of Clinical Chemistry, Georg-August-University Göttingen, Germany; 4 Department of Psychosomatic Medicine and Psychotherapy, Georg-August-University Göttingen, Germany

## Abstract

**Background:**

Screening of primary care patients at risk for left ventricular systolic dysfunction by a simple blood-test might reduce referral rates for echocardiography. Whether or not natriuretic peptide testing is a useful and cost-effective diagnostic instrument in primary care settings, however, is still a matter of debate.

**Methods:**

N-terminal pro-brain natriuretic peptide (NT-proBNP) levels, clinical information, and echocardiographic data of left ventricular systolic function were collected in 542 family practice patients with at least one cardiovascular risk factor. We determined the diagnostic power of the NT-proBNP assessment in ruling out left ventricular systolic dysfunction and compared it to a risk score derived from a logistic regression model of easily acquired clinical information.

**Results:**

23 of 542 patients showed left ventricular systolic dysfunction. Both NT-proBNP and the clinical risk score consisting of dyspnea at exertion and ankle swelling, coronary artery disease and diuretic treatment showed excellent diagnostic power for ruling out left ventricular systolic dysfunction. AUC of NT-proBNP was 0.83 (95% CI, 0.75 to 0.92) with a sensitivity of 0.91 (95% CI, 0.71 to 0.98) and a specificity of 0.46 (95% CI, 0.41 to 0.50). AUC of the clinical risk score was 0.85 (95% CI, 0.79 to 0.91) with a sensitivity of 0.91 (95% CI, 0.71 to 0.98) and a specificity of 0.64 (95% CI, 0.59 to 0.67). 148 misclassifications using NT-proBNP and 55 using the clinical risk score revealed a significant difference (McNemar test; p < 0.001) that was based on the higher specificity of the clinical risk score.

**Conclusion:**

The evaluation of clinical information is at least as effective as NT-proBNP testing in ruling out left ventricular systolic dysfunction in family practice patients at risk. If these results are confirmed in larger cohorts and in different samples, family physicians should be encouraged to rely on the diagnostic power of the clinical information from their patients.

## Background

Early detection of left ventricular systolic dysfunction is important, since early treatment with ACE inhibitors has been shown to delay the progression toward overt congestive heart failure (CHF) and to prolong life [1]. Since diagnosis of left ventricular systolic dysfunction solely based on clinical symptoms may be difficult [2], echocardiography is recommended as the diagnostic gold standard [3]. However, access to echocardiography in a primary care setting is limited, as the European survey of primary care physician perceptions on heart failure diagnosis and management (EURO-HF) showed [4].

To date, general practitioners have to identify patients in need of a referral to echocardiography by clinical information, which is routinely obtained from medical history and physical examination. Eventually, a pre-selection of patients at risk could reduce referral rates for echocardiography [3]. Therefore, a screening test (such as blood natriuretic peptide concentrations) which could easily be administered in primary care and reliably rule out left ventricular systolic dysfunction would reduce referral rates for echocardiography and lower health care costs. A retrospective analysis of cost-effectiveness showed that brain natriuretic peptide (BNP) testing could have reduced the costs per detected case by 26% compared to echocardiography [5].

Recent studies showed that the assessment of patients at high risk for left ventricular systolic dysfunction by means of N-terminal pro-brain natriuretic peptide (NT-proBNP) assays was valuable in the diagnosis of left ventricular systolic dysfunction [6,7] and heart failure [8]. However, there are conflicting results as to the usefulness of natriuretic peptides in identifying left ventricular systolic dysfunction after myocardial infarction [9]. Thus, ruling out left ventricular systolic dysfunction in primary care patients at risk by NT-proBNP is still a matter of debate. Additionally, the question whether NT-proBNP is diagnostically more suitable than easily available clinical information (as used today) has not yet been examined.

We therefore investigated the diagnostic ability of NT-proBNP testing compared to a risk score derived from a logistic regression model of easily acquired clinical information to detect left ventricular systolic dysfunction in primary care patients at risk.

## Methods

### Study Population

Between January 2003 and June 2004, 2,273 primary care patients from 58 practices in the city of Göttingen (North Germany) and the surrounding communities were invited by their general practitioners to participate in the study. Inclusion criteria were the presence of at least one cardiovascular risk factor documented by the family physician, including arterial hypertension, diabetes, family history of early heart disease, and coronary artery disease. Patients were classified as hypertensive if hypertension was documented by their treating physician or if they were on antihypertensive therapy. Patients were classified as diabetic if this diagnosis was made by their treating physician or if they were on antihyperglycemic therapy.

Exclusion criteria were the diagnosis of heart failure documented by the family physician or the presence of a terminal or disabling chronic disease. Patients received a leaflet with general information about the purpose of the study and how to participate. A total of 542 patients agreed to participate and were examined at the Georg-August-University, department of cardiology. Patients were interviewed, clinically examined, and assessed by echocardiography. Fifteen ml of blood were taken from a forearm vein for the measurement of NT-proBNP level.

### Determination of N-terminal brain natriuretic peptide

Before the study appointment, patients completed an overnight fast except for taking their regularly prescribed medications. A 21-gauge butterfly needle was inserted intravenously in the forearm, and after a 30-minute supine rest, blood samples were drawn into lithium-heparinate tubes; these were centrifugated and the supernatant was divided into aliquots and stored at minus 70°C. We used the Elecsys^® ^assay (Roche Diagnostics, Mannheim, Germany) to measure NT-proBNP in defrosted samples. The lowest detectable measurement for this assay was 5 pg/mL. The interassay coefficient of variation was 2.7% for 175 pg/mL, 2.7% for 355 pg/mL, 1.9% for 1068 pg/mL and 1.8% for 4962 pg/ml. The laboratory technician who measured NT-proBNP levels was at a different site and blinded to the characteristics of the patients and the results of echocardiograms. NT-proBNP reagents were kindly provided by Roche Diagnostics (Professor G. Hess, Mannheim, Germany).

### Echocardiographic measurements

Echocardiograms were performed with a Phillips Ultrasound System (Phillips Sonos Agilent) using a 3.5-MHz transducer with the patient in the left lateral position. A complete resting 2D echocardiogram and Doppler ultrasound examination was performed. We obtained standard 2D parasternal long and short-axis to determine left ventricular dimensions. We calculated left ventricular ejection fraction (EF) by the quantitative 2-D (biplane Simpson) method. An EF<50% was defined as systolic ventricular dysfunction. Echocardiograms were additionally rated concerning abnormalities defined as diastolic dysfunction [10]. Three of the authors (CL, SK, RW) interpreted all echocardiograms and were blinded to the results of the NT-proBNP assay as well as to details of the medical history.

### Statistical analysis

Assessing group differences, we used t-tests for continuous variables and Chi-Square-tests for comparisons of frequencies. A multiple logistic regression analysis was done to identify clinical variables that have a statistically significant diagnostic value in predicting left ventricular systolic dysfunction. We used a backward conditional model, including sociodemographic and clinical variables. Selected variables were used to estimate an individual patient risk score, thus, the sum of the β coefficients for each of the specific risk factors multiplied by their actual values [11].

We assessed the diagnostic performance of the NT-proBNP assay and the risk score by using receiver operating characteristic curves (ROC). The overall discriminative power of NT-proBNP and the clinical risk score is shown by the area under the curve (AUC). Comparisons between AUCs were assessed according to the method of Hanley & McNeil [12]. In addition to the AUC, we compared the test accuracy of NT-proBNP and the clinical risk score using the McNemar test (i.e. comparison of discordant pairs of false classifications). We chose cut-off points that gave comparable high sensitivity levels and moderate (at least 40%) specificity levels in order to optimize the negative predictive power of the test (SnNout; very high sensitivity: negative result rules out the diagnosis/disease) [13,14].

All analyses were two-tailed and the alpha was defined at 0.05. Statistical analyses were carried out using SPSS and Microsoft EXCEL. The study was approved by the local ethics committee, and all patients gave written informed consent before examination.

## Results

### Left ventricular function in patients at high risk for heart failure

Table 1 shows the sociodemographic and clinical characteristics of the 542 patients. In 23 patients (4%), EF was below 50%. These patients were significantly older, and complained significantly more often about dyspnea and ankle swelling than those with an EF ≥ 50%. Patients with a reduced EF also reported more often a history of myocardial infarction, coronary artery disease, and hyperlipidemia, and more often took diuretics and lipid-lowering agents. Levels of NT-proBNP were significantly higher in patients with systolic dysfunction when compared to patients with preserved left ventricular function. Three hundred and ninety nine patients of those without systolic dysfunction showed signs of diastolic dysfunction; in most cases (83%) of the lowest grade, i.e. impaired relaxation.

**Table 1:** Demographic and clinical characteristics, and drug treatment of patients with preserved and with impaired left ventricular systolic function.

Variables^†^	Patients with preserved systolic function (n = 519)	Patients with impaired systolic function (n = 23)	P*
Patient characteristics			
Age, *years*,	63 (62 to 63)	69 (66 to 73)	0.003
Male, %	57	70	0.241
BMI	29 (29 to 30)	29 (27 to 31)	0.854
Systolic BP, *mm Hg*	151 (149 to 152)	146 (138 to 155)	0.342
Diastolic BP, *mm Hg*	86 (85 to 86)	83 (78 to 89)	0.361
Ejection Fraction, %	61 (60 to 62)	41 (38 to 45)	<0.001
NT-proBNP, *pg/ml*	218 (174 to 259)	1154 (236 to 2072)	<0.001
Symptoms			
Dyspnea at exertion, %	34	74	<0.001
Dyspnea at rest, %	1	9	0.003
Ankle swelling, %	33	74	<0.001
Medical history			
Diabetes, %	31	39	0.400
Hypertension, %	86	96	0.184
Hyperlipidemia, %	51	74	0.032
CAD, %	29	65	<0.001
Myocardial infarction, %	7	22	0.019
Fam. history heart disease, %	42	57	0.168
Drug treatment			
Diuretics, %	42	74	0.003
β blockers, %	57	74	0.105
Calcium channel blockers, %	23	22	0.928
ACE inhibitors, %	46	52	0.564
AT1 blockers, %	16	26	0.201
Lipid lowerings agents, %	35	74	<0.001

* *T*-Test or χ^2^-Test.

^† ^values in mean (95% confidence interval) unless otherwise indicated.

BMI Body Mass Index; BP blood pressure; CAD coronary artery disease; ACE angiotension converting enzyme, AT1 blocker angiotensin II type 1 receptor blocker.

### Relationship of left ventricular systolic dysfunction with sociodemographic and clinical variables

A logistic stepwise regression with left ventricular dysfunction as the dependent variable and 17 independent variables as covariates was conducted. The backward conditional model selected the following three covariates: dyspnea at exertion combined with ankle swelling, history of coronary artery disease, and treatment with diuretics. Other variables were entered into the model but were excluded by the stepwise regression: age (dichotomized by the median), sex, BMI>30, diabetes mellitus, hypertension, hyperlipidemia, myocardial infarction, family history of early heart disease, treatment with ACE inhibitors, β blockers, calcium channel blockers, AT1 blockers, or lipid lowering agents (Table 2).

**Table 2:** Logistic regression analysis of demographic and clinical variables associated with left ventricular systolic dysfunction.*

Covariates	Regression coefficient	Odds ratio	(95% CI)
Dyspnea at exertion + ankle swelling	1.819	6.165	(2.400 to 15.842)
Coronary artery disease	1.182	3.261	(1.282 to 8.293)
Diuretic treatment	1.035	2.814	(1.038 to 7.626)

*Presented covariates were selected by backward conditional model. Other variables entered into model were age>64 years, sex, BMI>30, diabetes mellitus, hypertension, hyperlipidemia, myocardial infarction; family history of early heart disease, dyspnea at rest, taking ACE inhibitors, β blockers, calcium channel blockers, AT1 blockers, lipid lowering agents.

### Diagnoses of left ventricular systolic dysfunction based on NT-proBNP levels or clinical information

**Figure 1:**
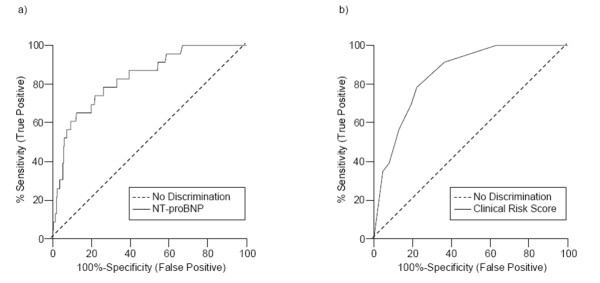
Receiver-operator characteristic (ROC) curves for (a) NT-proBNP (AUC = 83%; 95 % CI = 75% to 92%) and (b) clinical risk score (AUC = 85%; 95 % CI = 79% to 91%) in the diagnosis of left ventricular systolic dysfunction confirmed by echocardiography in patients with preserved (n = 519) and impaired (n = 23) left ventricular function.

Figure 1a shows the AUC for NT-proBNP. This illustrates its diagnostic value for left ventricular systolic dysfunction, which can be rated as excellent. The cut-off point of NT-proBNP was determined as having a moderate specificity and a high sensitivity compared to the clinical risk score. Concentrations below the cut-off point allowed for the exclusion of left ventricular systolic dysfunction. The negative likelihood ratio of a NT-proBNP value below 98.5 pg/ml is 0.19. It indicates that the probability of a negative test result is five times lower in patients with impaired left ventricular systolic dysfunction than in patients with preserved left ventricular function. Table 3 shows the cut-off point dependent measures of NT-proBNP as a diagnostic test.

**Table 3:** Test accuracy of NT-proBNP and the clinical risk score for identifying left ventricular systolic dysfunction.*

	True Positive	False Positive	False Negative	True Negative	Sensitivity (%)	Specificity (%)	Likelihood ratio of negative result (95% CI)
NT-proBNP (< 98.5 pg/ml)	21	282	2	237	91 (71 to 98)	46 (41 to 50)	0.19 (0.05 to 0.71)
Risk score (< 1.11)	21	189	2	330	91 (71 to 98)	64 (59 to 67)	0.14 (0.04 to 0.51)

*At cut-off points with comparable high sensitivity and moderate specificity in order to rule out left ventricular dysfunction in case of a negative result (SnNout; very high sensitivity: negative result rules out the diagnosis/disease. Values in parentheses are 95% confidence intervals.

Figure 1b shows the AUC for the clinical risk score including the following variables: suffering from ankle swelling and dyspnea at exertion, history of coronary artery disease, and treatment with diuretics. The backward conditional model with left ventricular dysfunction as the dependent variable selected these risk factors (Table 2). To obtain the individual patient risk score, the β coefficients for each of the specific risk factors were multiplied by their actual values and then summed up. For example, individual patient risk score = (β_1 _× dyspnea at exertion × ankle swelling) + (β_2 _× coronary artery disease) + (β_3 _× diuretics). AUC of the clinical risk score indicates its diagnostic value for left ventricular systolic dysfunction and can be rated as excellent.

The cut-off point was determined as having a moderate specificity and a high sensitivity compared to the NT-proBNP test. The negative likelihood ratio of a clinical risk score below 1.11 is 0.14 and indicates that the probability of a negative test result is seven times lower in patients with impaired left ventricular systolic dysfunction than in patients with preserved left ventricular function. Table 3 shows the cut-off point dependent measures for the clinical risk score as a diagnostic test.

### Comparison of NT-proBNP testing and the clinical risk score for identifying left ventricular systolic dysfunction

The minimal difference of 0.019 between AUCs of NT-proBNP and the clinical risk score was not significant (Figure 1). Table 4 shows the number of correct and false classifications for both, the risk score and NT-proBNP as single diagnostic tests. Comparing the two tests, there are four different cases possible: Cases shown in the first line and first row (n = 203) were correctly classified by both tests, i.e. they are true positive and true negative diagnoses. Cases shown in the second line and second row (n= 136) were falsely classified by both tests, i.e. they are false positive and false negative diagnoses. Cases shown in the first line and second row (n = 148) were falsely classified by NT-proBNP but correctly classified by the risk score. Cases shown in the second line and first row (n = 55) were falsely classified by the risk score but correctly classified by NT-proBNP. The McNemar test showed that this difference concerning the discordant pairs of false classifications was significant. The advantage of using the clinical risk score for classification was based on the better specificity, resulting in a lower rate of false positive cases.

**Table 4:** Results of classification of patients with impaired left ventricular function by NT-proBNP and risk score at cut-off points with high sensitivity and moderate specificity.

	NT-proBNP correct classification	NT-proBNP false classification
Risk score correct classification	203	148
Risk score false classification	55	136

McNemar test: p < 0.001.

## Discussion

To our knowledge, this study is the first to compare the diagnostic power of NT-proBNP assessment and a risk score derived from a logistic regression model of easily acquired clinical information in a primary care setting. Both, NT-proBNP testing and the clinical risk score reliably ruled out left ventricular systolic dysfunction in a cohort of 542 primary care patients at risk. This data confirmed the diagnostic value of NT-proBNP assessment in ruling out left ventricular systolic dysfunction [15,16].

Although we aimed for a large number of patients to participate in the study, only 542 of 2273 patients accepted the invitation and could be included. Thus, our study has some limitations. First, the sample size of our study is too small to derive age and gender adjusted cut-off values that might have improved the diagnostic accuracy of NT-proBNP. Additionally, our logistic regression might have reduced statistical evidence due to the high number of independent variables. As we did not perform a hypothesis testing regression analysis we assume that it was suitable for development of our clinical risk score. Second, our study may be biased to model a reliable and valid risk equation and therefore it is necessary for further studies to recalculate our clinical risk score equation in larger cohorts and evaluate it in different samples [17]. Third, we decided not to take the echocardiographic findings of diastolic dysfunction into account for statistical analysis, since there are divergent study findings concerning the clinical implication of the diagnosis of diastolic dysfunction in primary care. Critical objections affect the lack of evidence based treatment [18] and poor concordance of echocardiographic measures [19]. Fourth, our study might have been further improved by using an electrocardiography as an additional diagnostic tool since its usefulness in detecting left ventricular systolic dysfunction has been suggested [20,21]. However, due to a recent meta-analysis electrocardiography may not be used for ruling out left ventricular hypertrophy in patients with hypertension [22].

We used a logistic regression analysis to model the risk score including demographic and clinical variables. Independent factors significantly associated with systolic dysfunction were suffering from dyspnea at exertion and ankle swelling, a history of coronary artery disease, and taking diuretics. In a comparable study of 764 primary care patients, Raymond et al. conducted a logistic regression analysis and revealed male sex, admission for pulmonary congestion and/or myocardial infarction, and breathlessness as significant factors to predict left ventricular systolic dysfunction [21]. Although our study showed that history of myocardial infarction and prescription of diuretics and lipid lowering agents was more frequent in patients with impaired than with preserved systolic function (see table 1), this variables were not selected by the regression analysis. In contrast to Raymond et al. our study sample did not reveal any difference in the distribution of male and female patients concerning systolic function (see table 1). An important difference between the two studies concerned the definition of left ventricular systolic dysfunction: Raymond et al. defined left ventricular systolic dysfunction as EF<40%, so cases of less severe left ventricular systolic dysfunction were treated as unimpaired. It might be speculated, that this caused the different results.

Heidenreich et al. concluded that screening with BNP followed by echocardiography is economically attractive for patient groups with at least a 1% prevalence of left ventricular systolic dysfunction [23]. Our study confirmed a 4.3% prevalence for left ventricular systolic dysfunction in a primary care setting using echocardiography. Therefore, NT-proBNP assessment (which has the same assay costs as BNP) would have been highly cost-effective. Irrespective of its diagnostic power as a screening instrument, however, the additional costs of natriuretic peptide testing is not self-evidently justified by its test accuracy [24]. In our study, the risk score derived from a logistic regression model of easily acquired clinical information would have been even more cost-effective than assessing NT-proBNP, because all information is available without any additional examination [25]. Such a risk score solely based on clinical information would offer every general practitioner direct access to a diagnostic tool with a test accuracy as excellent as NT-proBNP testing. The multivariate logistic regression analysis revealed three easily established factors as significant covariates. Since risk factors seldom occur in isolation, modelling a risk score by using a set of established risk factors is essential [26]. High values of NT-pro BNP predict mortality even better than the presence of left ventricular systolic dysfunction does [27]. However, treatment with ACE inhibitors is recommended for patients with left ventricular systolic dysfunction but not for patients with isolated elevated levels of NT-pro BNP.

Given the hypothesis that all patients of our study had been considered for echocardiography, the clinical risk score would have reduced the number of referrals to echocardiography from 542 (all cases) to 210 (cases where left ventricular systolic dysfunction could not be ruled out), with 21 true positives and 189 false positives. That means, 93 more cases could have been ruled out with the clinical risk score than with NT-proBNP assessment, which would have reduced the number of referrals from 542 to 303, with 21 true positives and 282 false positives.

## Conclusion

According to our comparison, an evaluation of clinical information could be at least as effective as NT-proBNP testing in ruling out left ventricular systolic dysfunction in primary care patients at risk. Therefore, general practitioners should be encouraged to rely on the diagnostic power of the clinical information their patients provide.

## Competing interests

Roche Diagnostics (Professor G. Hess, Mannheim, Germany) was an official member of the study group and supported the present investigation with NT-proBNP test kits. There are no other competing interests.

## Authors' contributions

JK and MS performed statistical analyses, wrote and edited the manuscript; DW recruited the patients and coordinated the study; CL, SK, and RW performed the clinical and echocardiographic assessment; CP supervised the measurement of the NT-proBNP levels; MMK, CHL, BP, and LB led conception and design; all authors have contributed to the conception and design and revised the manuscript. All authors read and approved the final manuscript.

## Pre-publication history

The pre-publication history for this paper can be accessed here:



## References

[B1] The SOLVD Investigators (1992). Effect of enalapril on mortality and the development of heart failure in asymptomatic patients with reduced left ventricular ejection fractions. N Engl J Med.

[B2] Badgett RG, Lucey CR, Mulrow CD (1997). Can the clinical examination diagnose left-sided heart failure in adults?. JAMA.

[B3] Hunt SA, Abraham WT, Chin MH, Feldman AM, Francis GS, Ganiats TG, Jessup M, Konstam MA, Mancini DM, Michl K, Oates JA, Rahko PS, Silver MA, Stevenson LW, Yancy CW, Antman EM, Smith SC, Adams CD, Anderson JL, Faxon DP, Fuster V, Halperin JL, Hiratzka LF, Jacobs AK, Nishimura R, Ornato JP, Page RL, Riegel B, American College of Cardiology; American Heart Association Task Force on Practice Guidelines; American College of Chest Physicians; International Society for Heart and Lung Transplantation; Heart Rhythm Society (2005). ACC/AHA 2005 guideline update for the diagnosis and management of chronic heart failure in the adult: a report of the American College of Cardiology/American Heart Association Task Force on Practice Guidelines (writing committee to update the 2001 guidelines for the evaluation and management of heart failure). Circulation.

[B4] Hobbs FDR, Jones MI, Allan TF, Wilson S, Tobias R (2000). European survey of primary care physician perceptions on heart failure diagnosis and management (EURO-HF). Eur Heart J.

[B5] Nielsen OW, McDonagh TA, Robb SD, Dargie HJ (2003). Retrospective analysis of the cost-effectiveness of using plasma brain natriuretic peptide in screening for left ventricular systolic dysfunction in the general population. JACC.

[B6] Groenning BA, Raymond I, Hildebrandt JC, Nilsson JC, Baumann M, Pedersen F (2004). Diagnostic and prognostic evaluation of left ventricular systolic heart failure by plasma N-terminal pro-brain natriuretic peptide concentrations in a large sample of the general population. Heart.

[B7] Gustafsson F, Steensgaard-Hansen F, Badskjaer J, Poulsen AH, Corell P, Hildebrandt P (2005). Diagnostic and prognostic performance of NT-terminal proBNP in primary care patients with suspected heart failure. J Card Fail.

[B8] Hobbs FDR, Davis RC, Roalfe AK, Hare R, Davies MK, Kenkre AK (2002). Reliability of N-terminal pro-brain natriuretic peptide assay in diagnosis of heart failure. cohort study in representative and high risk community populations. BMJ.

[B9] McClure SJ, Caruana L, Davie AP, Goldthorp S, McMurray JJV (1998). Cohort study of plasma natriuretic peptides for identifying left ventricular systolic dysfunction in primary care. BMJ.

[B10] Lubien E, DeMaria A, Krishnaswamy P, Clopton P, Koon J, Kazanegra R, Gardetto N, Wanner E, Maisel AS (2002). Utility of B-natriuretic peptide in detecting diastolic dysfunction. Comparison with Doppler velocity recordings. Circulation.

[B11] Kannel WB, D'Agostino RB, Silbershatz H, Belanger AJ, Wilson PWF, Levy D (1999). Profile for estimating risk of heart failure. Arch Intern Med.

[B12] Hanley JA, McNeil BJ (1983). A method of comparing the areas under receiver operating characteristic curves derived from the same cases. Radiology.

[B13] Sackett DL, Haynes RB (2002). The architecture of diagnostic research. BMJ.

[B14] Pewsner D, Battaglia M, Minder C, Marx H, Bucher HC, Egger M (2004). Ruling a diagnosis in or out with "SpPIn" and "SnNOut": a note of caution. BMJ.

[B15] Battaglia M, Pewsner D, Jüni P, Egger M, Bucher HC, Bachmann LM (2006). Accuracy of B-type natriuretic peptide tests to exclude congestive heart failure. Systematic review of test accuracy studies. Arch Intern Med.

[B16] Doust JA, Glasziou P, Pietrzak E, Dobsen A (2004). A systematic review of the diagnostic accuracy of natriuretic peptides for heart failure. Arch Intern.

[B17] Cowie MR (2003). Estimating prognosis in heart failure: time for a better approach. Heart.

[B18] Caruana L, Petrie MC, McMurray JJV (2000). Do patients with suspected heart failure and preserved left ventricular systolic function suffer from "diastolic heart failure" or from misdiagnosis? A prospective descriptive study. BMJ.

[B19] Petrie MC, Hogg K, Caruana L, McMurray JJV (2004). Poor concordance of commonly used echocardiographic measures of left ventricular diastolic dysfunction in patients with suspected heart failure but preserved systolic function: is there a reliable echocardiographic measure of diastolic dysfunction?. Heart.

[B20] Nielsen OW, Hansen JF, Hilden J, Larsen CT, Svanegaard J (2000). Risk assessment of left ventricularsystolic dysfunction in primary care: cross sectional study evaluating a range of diagnostic tests. BMJ.

[B21] Raymond I, Pedersen F, Steensgaard-Hansen F, Green A, Busch-Sorensen M, Tuxen C, Appel J, Jacobsen J, Atar D, Hildebrandt P (2003). Prevalence of impaired left ventricular systolic function and heart failure in a middle aged and elderly urban population segment of Copenhagen. Heart.

[B22] Pewsner D, Jüni P, Egger M, Battaglia M, Sundström J, Bachmann LM (2007). Accuracy of electrocardiography in diagnosis of left ventricular hypertrophy in arterial hypertension: systematic review. BMJ.

[B23] Heidenreich PA, Gubens MA, Fonarow GC, Konstam MA, Stevenson LW, Shekelle PG (2004). Cost-effectiveness of screening with B-type natriuretic peptide to identify patients with reduced left ventricular ejection fraction. JACC.

[B24] Davenport C, Cheng EY, Kwok YT, Lai AH, Wakabayashi T, Hyde C, Connock M (2006). Assessing the diagnostic test accuracy of natriuretic peptides and ECG in the diagnosis of left ventricular systolic dysfunction: a systematic review and meta-analysis. Br J Gen Pract.

[B25] Landray MJ, Lehman R, Arnold I (2000). Measuring brain natriuretic peptide in suspected left ventricular systolic dysfunction in general practice: cross-sectional study. BMJ.

[B26] Kannel WB, D'Agostino RB, Sullivan L, Wilson PWF (2004). Concept and usefulness of cardiovascular risk profiles. Am Heart J.

[B27] Rutten JH, Boomsma F, Van den Meiracker AH (2007). Diagnostic and prognostic value of B-type natriuretic peptides in heart failure or signs of heart failure. Ned Tijdschr Geneeskd.

